# The Effect of Stress, Anxiety and Burnout Levels of Healthcare Professionals Caring for COVID-19 Patients on Their Quality of Life

**DOI:** 10.3389/fpsyg.2020.597624

**Published:** 2020-11-23

**Authors:** Nuriye Çelmeçe, Mustafa Menekay

**Affiliations:** Department of International Business, Near East University, Nicosia, Cyprus

**Keywords:** stress, anxiety, burnout, quality of life, healthcare professionals

## Abstract

**Background:**

The healthcare system is among the institutions operating under the most challenging conditions during the period of outbreaks like pandemic which affects the whole world and leads to deaths. During pandemics that affect the society in terms of socioeconomic and mental aspects, the mental health of healthcare teams, who undertake a heavy social and work load, is affected by this situation.

**Aim:**

This research was conducted with the aim of determining the effect of stress, anxiety, and burnout levels of healthcare professionals (doctors, nurses, healthcare assistants) caring for COVID-19 patients on their quality of life.

**Method:**

The sample of the study consisted of a total of 240 healthcare professionals, determined by random sampling method among the healthcare professionals working in pandemic hospitals in Tokat city center, Turkey. Perceived Stress Scale, Spielberger State-Trait Anxiety Inventory, Maslach Burnout Inventory and Quality of Life Scale were used in the study conducted in the relational screening model.

**Findings:**

While the stress, trait anxiety, and quality of life mean scores of healthcare professionals who were female, married and had children were higher than the other groups, high, moderate, negative, and positive correlations were found among all variables.

**Conclusion:**

The research concluded that the stress, anxiety, and burnout of healthcare workers caring for COVID-19 patients affected their quality of life.

## Introduction

COVID-19, which was declared to be a pandemic by the World Health Organization (WHO) after emerging in Wuhan, China, has had an impact on many countries other than the point where it started to spread and mostly affected the healthcare professionals. Healthcare professionals are at risk of exposure to infectious diseases, including the novel coronavirus (COVID-19) that emerged in China. Working face-to-face with people who are quarantined and carrying the virus can raise serious concerns such as fear of death among healthcare professionals, feelings of loneliness and anger may develop, and these emotions may lead to stress in the professionals (Xiang et al., 2020; Zhang et al., 2020). In a study conducted by Cai et al. (2020) with 534 doctors, nurses and primary care providers in Hubei state, healthcare professionals’ stress levels were found to be extremely high during the COVID-19 outbreak. As a result of a study conducted in Turkey, it was determined that 38% of nurses working in the emergency department experienced stress. According to the same research, it was revealed that nurses experiencing stress regretted their choice of profession and thought to quit or leave their jobs (Yasal and Partlak, 2019). The study conducted by Jiang et al. (2020) with 310 people in order to examine the psychological symptoms of healthcare professionals in Wuhan during the COVID-19 outbreak showed that healthcare professionals were under moderate to severe stress, and that many of them reported high anxiety and depression. Emergencies such as the COVID-19 outbreak can prove riskier for healthcare professionals who work under various difficulties and risks even in the provision of ordinary health services. These situations can cause them to be exposed to severe stress reactions and experience stress, which increases the risk of developing secondary trauma.

Although stress, fatigue, and increased workload can have various consequences such as musculoskeletal disorders, one of the most likely negative consequences of these conditions is increased burnout in healthcare. It is known that the risk of getting the virus, which spreads gradually during the epidemic, increases the anxiety of getting sick both for themselves and their relatives and can lead to burnout in carrying out tasks that require endurance. During epidemic periods such as COVID-19, the most important causes of burnout are prolonged working hours, the anxiety of getting sick with increasing virus load, and most importantly, all kinds of discourses and approaches that devalue the labor of healthcare professionals who risk their own health. Burnout is mostly seen in those who work in professions such as “health,” which directly serve people and where human relations are important. In studies conducted outside the epidemic period, high levels of burnout have been reported among healthcare professionals both in our country and in the world (Erol et al., 2012; Kansoun et al., 2019). In the study of Matsuo et al. (2020), where they investigated the prevalence of burnout in healthcare professionals during the COVID-19 outbreak in Japan with the participation of 488 healthcare professionals, the overall burnout prevalence was found to be 31.4% (98 out of 312). 59 (46.8%) of 126 nurses, 8 (36.4%) of 22 radiology technologists, and 7 (36.8%) of 19 pharmacists were experiencing burnout.

A study conducted on healthcare professionals showed a significant positive relationship between stress and burnout (Morgantini et al., 2020). Another study investigating the relationship between burnout, anxiety, and stress disorders during the COVID-19 epidemic reported that doctors and nurses experienced high levels of mental health problems, including burnout (Sung et al., 2020).

The stress and anxiety and burnout of physicians, nurses and assistant healthcare personnel who are in direct contact with patients can affect both their job performance and health conditions and reduce their quality of life. In healthcare professionals, the anxiety that occurs during or as a result of crisis intervention may impair their mental reasoning and abstract thinking skills and cause a lack of attention and coordination. Various emotions such as fear and anxiety can affect their problem-solving performance. The decrease in problem-solving ability may lead to a decrease in the efficiency of the services provided to protect the health of individuals and public health and to facilitate living conditions.

In this study, it was aimed to determine the effect of stress, anxiety, and burnout levels of healthcare professionals who work actively in hospitals during the COVID-19 pandemic on their quality of life. In line with this aim, answers to the following questions were sought:

1. Is there a relationship between the stress, anxiety, and burnout levels of healthcare professionals and their quality of life?
2. Do stress, anxiety, and burnout levels of healthcare professionals have an impact on their quality of life?

## Materials and Methods

The research was conducted in the relational screening model in order to determine the effect of stress, anxiety, and burnout levels of healthcare professionals on their quality of life. This cross-sectional study was conducted between 20 May 2020 and 10 June 2020 by applying an anonymous online survey to HCWs who were actively working during the COVID-19 pandemic. All subjects provided informed consent electronically before registration. Only subjects who agreed to participate voluntarily were included in this study, and subjects could quit the process at any time. Only one response per person to the questionnaire was permitted. Incomplete surveys were not included in this study. The target population of the study consists of healthcare professionals working in pandemic hospitals in the city center of Tokat. While determining the sampling method, the simple random sampling technique, which is the sampling type in which all the elements in the study population have an equal chance to be selected, was used. For the sample size, the “disproportionate element sampling” technique was used. A total of 240 healthcare workers, 170 women (70%), and 70 men (30%) participated in the study.

The desired number of the population could not be reached as a result of healthcare professionals having to work hard due to the pandemic, being on leave, or being excused for reasons such as illness or birth. Variable types used in the analysis are limited by the reliability of the applied survey. The fact that some hospital staff did not participate in the survey made the sample smaller.

### Measurement Tools

#### Perceived Stress Scale

The scale, developed by Cohen et al. (1983) (Cronbach’s alpha = 0.78) and adapted to Turkish by Eskin et al. (2013) (Cronbach’s alpha = 0.84), consists of 14 items. The scale was developed in order to measure the degree to which situations in one’s life are appraised as stressful (Eskin et al., 2013). Participants are asked to rate the items on a 5-point Likert-type scale (Never = 0; Very often = 4). The scores of 7 items with positive statements are reversed. The total score ranges from 0 to 56, and a high score indicates the person’s excessive perceived stress.

#### Spielberger State-Trait Anxiety Inventory

The scale was developed by Spielberger et al. in 1964 in order to determine the trait anxiety levels of individuals. The Turkish reliability and validity studies of the scale were conducted by Öner and Le Compte (1983). Trait anxiety scale measures anxiety in adolescents over the age of 14 and adults. This scale, which is a kind of self-assessment scale, includes 20 items consisting of short expressions. All items are scored between 1 and 4 by reversing the scores in the reverse items included in the scale, and the increasing score indicates high level of anxiety. The total score obtained from the scale can vary between 20 and 80. A high score indicates high level of anxiety, while a low score indicates low level of anxiety.

#### Maslach Burnout Scale

Maslach Burnout Scale was developed by Maslach and Jackson (1981) in order to determine the level of burnout. It is a scale consisting of three sub-dimensions as emotional exhaustion, depersonalization, and personal accomplishment and 22 items that have defining characteristics of these dimensions. The Cronbach’s α coefficients of the sub-dimensions of the scale, which was adapted into Turkish by Ergin (1992), were found as 0.86 for the emotional exhaustion sub-dimension, 0.71 for the depersonalization sub-dimension, and 0.76 for the personal accomplishment sub-dimension.

#### Quality of Life Scale

It was developed by Menekay and Çelmeçe (2017) in order to determine employees’ attitudes toward their quality of life. The scale consists of 28 items and four dimensions: working life, social life, burnout, and satisfaction. The reliability coefficients of the sub-dimensions of the scale vary between 0.637 and 0.831. The reliability coefficient of the whole scale is 0.881 (Menekay and Çelmeçe, 2017).

### Measurement Model

In the reliability and validity analysis, the first point to be considered about variables is internal consistency reliability. Internal consistency reliability is commonly measured with Cronbach’s Alpha value. The second thing to look at with regard to variables is validity. The validity check is done in two stages as convergent validity and discriminant validity. Two values are considered for merger validity (Doğan, 2019). These are the outer loadings and the explained average variance extracted (AVE) values. There are three methods recommended for divergence validity (Doğan, 2019). The first of these is the Fornell and Larcker criterion (Hair et al., 2017), the second is cross loadings values, and the third is the HTMT (Heterotrait-Monotrait Ratio) criterion (Henseler et al., 2015). According to the Fornell and Larcker (1981) criterion, the square root of the AVE values of the structures included in the study should be higher than the correlations between the structures included in the study. Table 1 shows the results of the analysis made according to the Fornell and Larcker (1981) criterion. According to the criterion of Henseler et al. (2015), HTMT (Heterotrait-Monotrait Ratio) expresses the ratio of the mean correlations of the expressions of all variables included in the study, to the geometric average of the correlations (the heterotrait-heteromethod correlations) of the expressions of the same variable. The authors stated that the HTMT value, 0.90, should be under 0.85 for concepts that are distant from each other in terms of content. HTMT values are given in Table 2.

**TABLE 1:** Divergence validity results (Fornell and larckell criterion).

Stress	Trait anxiety	Burnout	Quality of life	
Stress	**(0.912)**			
Trait anxiety	0.669	**(0.925)**		
Burnout	0.627	0.610	**(0.907)**	
Quality of life	0.678	0.705	0.721	**(0.892)**

*Significant values are written in bold.*

**TABLE 2:** Divergence validity results (HTMT criterion).

Stress	Trait anxiety	Burnout	Quality of life
Stress			
Trait anxiety	0.731		
Burnout	0.689	0.667	
Quality of life	0.747	0.766	0.790

The values in parentheses in the table are the square root values of AVE. When the values in the table are examined, it is seen that the explained mean the square root of the variance of each structure is higher than its correlation with other structures.

When the values in the table are examined, it is seen that the HTMT values are below the threshold value. Based on the findings in Tables 1, 2, it can be stated that the divergence validity was provided.

## Findings

A total of 240 people, 170 (70%) females and 70 (30%) males, participated in the study. The sociodemographic characteristics of the healthcare professionals participating in the study are presented in Table 3.

**TABLE 3:** Sociodemographic characteristics of the healthcare professionals participating in the study.

	*N*	%
**Gender**		
Female	170	70
Male	70	30
**Marital status**		
Married	130	54
Single	110	46
**Professional status**		
Doctor	70	30
Nurse	120	50
Assistant personnel	50	20
**Having children**		
Had children	114	47
Didn’t have children	126	53

It is seen among the healthcare professionals participating in the study that 70% of them were female, 30% were male, 54% were married, 46% were single, %30 were doctors, 50% were nurses, 20% were assistant personnel, 47% had children, and 53% did not have children.

The differences between the stress, trait anxiety, burnout, and quality of life mean scores of healthcare professionals are presented in Table 4.

**TABLE 4:** The differences between the stress, trait anxiety, burnout, and quality of life mean scores of healthcare professionals.

	Stress			Trait anxiety			Burnout			Quality of life		
	X	Ss		X	Ss		X	Ss		X	Ss	
**Gender**												
Female	67.5	13.3	*t* = −2.392	48.12	9.37	*t* = 3.214	30.1	9.24	*t* = 1.324	14.2	3.87	*t* = 0.731
Male	62.3	15.7	*p* = 0.017*	46.17	9.27	*p* = 0.001*	28.7	8.82	*p* = 0.122	13.8	3.14	*p* = 0.011*
**Marital status**												
Married	61.9	12.7	*t* = −2.380	44,21	9,54	*t* = 3.012	29.67	8.21	*t* = 1.789	22.61	4.41	*t* = 1.941
Single	56.4	13.8	*p* = 0.015*	40,31	9,21	*p* = 0.001*	28.22	6,98	*p* = 0.002*	19.87	4.23	*p* = 0.145
**Professionals status**												
Doctor	55	13.1	*t* = 1.301	45.98	9.56	*t* = 1.698	29.34	8.41	*t* = 2.564	14.29	3.65	*t* = 1.365
Nurse	62	14.7	*p* = 0.174	44.25	11.03	*p* = 0.081	26.98	8.91	*p* = 0.004*	13.62	3.56	*p* = 0.114
Assistant personnel	60	14.1		49.52	12.41		24.15	8.14		11.75	3.62	
**Having children**												
Had children	67.78	14.89	*t* = −1.876	47.23	9.97	*t* = 3.356	29.56	7.84	*t* = 1.623	49.11	8.44	*t* = 3.325
Didn’t have children	62.17	14.97	*p* = 0.013*	42.15	9.00	*p* = 0.001*	29.22	7.99	*p* = 0.121	44.29	9.45	*p* = 0.001*

*Significant values are written in bold. ^∗^*p* < 0.01.*

In Table 4, relationships between stress, trait anxiety, burnout, and quality of life scores and socio-demographic and other characteristics of healthcare professionals are observed.

The mean scores of stress (*t* = −2.392, *p* = 0.017, *X* = 67.5), trait anxiety (*t* = 3.214, *p* = 0.001, *X* = 48.12), and quality of life (*t* = 0.73, *p* = 0.011, *X* = 14.2) of female healthcare professionals were found to be significantly higher than those of men.

The mean scores of stress (*t* = −2.380, *p* = 0.015, *X* = 61.9), trait anxiety (*t* = 3.012, *p* = 0.001, *X* = 44.21), and burnout (*t* = 1.789, *p* = 0.002, *X* = 29.67) of married healthcare professionals were found to be significantly higher than those of single employees.

Considering the occupational status, the mean scores of burnout of the nurses (*t* = 2.564, *p* = 0.004, *X* = 8.91) were found to be significantly higher than the mean scores of doctors (*X* = 8.41) and other assistant personnel (*X* = 8.14).

The mean scores of stress (*t* = −1.876, *p* = 0.013, *X* = 67.78), anxiety (*t* = 3.356, *p* = 0.001, *X* = 9.97) and quality of life (*t* = 3.325, *p* = 0.001, *X* = 49.11) of healthcare professionals who had children were found to be significantly higher than those of employees who did not have children.

Findings regarding the correlations between the stress, anxiety, and burnout levels of healthcare professionals and their quality of life are presented in Table 5.

**TABLE 5:** Correlation values of the relationship between stress, trait anxiety, burnout levels, and quality of life of healthcare professionals.

		1	2	3	4
1.	Stress	–			
2.	Trait anxiety	0.64**	–		
3.	Burnout	0.46**	0.59**	–	
4.	Quality of life	−0.61**	−0.64**	−0.70**	–

*^∗∗^*p* < 0.01*

As seen in Table 5, high moderate positive correlations were found between stress and trait anxiety (*r* = 0.64; *p* < 0.001), burnout (*r* = 0.46; *p* < 0.001), and high and negative correlation was found between stress and quality of life (*r* = −0.61; *p* < 0.001). A moderate and positive correlation was found between trait anxiety and burnout (*r* = 0.59; *p* < 0.001) and a high and negative correlation was found between trait anxiety and quality of life (*r* = −0.64; *p* < 0.001). Again, a high and negative correlation was found between burnout and quality of life (*r* = −0.70, *p* < 0.001).

Multiple regression analysis was performed in order to test the effect of stress, trait anxiety, and burnout on quality of life, and the performed analyses are presented in Table 6.

**TABLE 6:** Regression values regarding the effect of stress, trait anxiety, burnout levels of healthcare professionals on their quality of life.

	β	*t*	*p*(sig)
Stress	−0.359	−3.887	0.000
Trait anxiety	−0.025	−0.292	0.000
Burnout	0.176	−1.837	0.000

When Table 6 is examined, it is possible to see that the *F*-value for stress is 11.470 at 0.00 significance value level and adjusted *R*^2^ is 0.196. Through the information obtained from the regression analysis, it is seen that stress, trait anxiety, and burnout explain the quality of life correlation variable by 19%. In addition, considering the *t*-statistics and *p*-values, it can be seen that there is a negative correlation between the independent variables and the dependent variable. This situation indicates that stress, anxiety, and burnout have a negative effect on the quality of life.

## Discussion

The coronavirus pandemic, which has deeply affected the world, has caused the death of thousands of people, and with the onset of the pandemic, healthcare professionals have had to work at a very busy pace. In our study, the effect of stress, trait anxiety, and burnout levels of healthcare professionals working during the COVID-19 pandemic on their quality of life was investigated.

Correlations between scores of stress, trait anxiety, burnout, and quality of life and socio-demographic and other characteristics of healthcare professionals were examined, and the mean scores of stress, trait anxiety, and quality of life of female healthcare professionals were found to be significantly higher than those of males. In the study conducted by Zhu et al. (2020) with 5,062 healthcare professionals in Tongji hospital in Wuhan, China, the stress scores of female employees were found to be higher. In the study conducted by Lai et al. (2020), stress levels of women were found to be high, and in another study conducted with employees in COVID-19 services in China, it was observed that nurses developed more psychological symptoms than physicians, and women developed more psychological symptoms than men (Huang et al., 2020). These findings support our study. According to the research findings, it was found that women experienced more work-related stress. This situation can be interpreted as women’s responsibilities related to social life and family outside of work cause them to be exposed to more stress. In the study conducted by Barello et al. (2020) with 376 healthcare professionals, the burnout levels of female healthcare workers were found to be higher than those of males.

The mean scores of stress, trait anxiety, and burnout of married healthcare professionals were found to be significantly higher than those of single employees. Working in a hospital where COVID-19 treatment is provided causes state anxiety levels. The increase in the weekly working hours of healthcare professionals increases the levels of anxiety and despair.

Considering the occupational status, the mean scores of burnout of the nurses were found to be significantly higher than the mean scores of doctors and other assistant personnel. In the study conducted by Hacimusalar et al. (2020), anxiety scores of nurses were found to be higher than other employees. This may be caused by the fact that nurses’ working conditions involve more negative changes compared to other healthcare professionals during the pandemic period. The fact that nurses have more physical contact with patients than doctors in inpatient wards may be an important factor. In a study conducted by Li et al. (2016) with nurses and doctors, it was reported that negative life events were associated with depression and anxiety symptoms, doctors experienced more work-related negative events than nurses, but nurses displayed higher levels of anxiety and depression (Li et al., 2016). In the study conducted by Barello et al. (2020) with 376 healthcare professionals, burnout levels of nurses were found to be higher than other healthcare professionals.

The mean scores of stress, anxiety, and quality of life of healthcare professionals who had children were found to be significantly higher than those of employees who did not have children. In the study conducted by Hacimusalar et al. (2020), the anxiety levels of those who had children were found to be higher. These findings support our study. Schools have been evacuated during the pandemic and the children have had to stay home. This situation has caused many problems for working parents such as the care and education of their children. Parents who cannot get permission from work since they have to work have had difficulty finding someone to take care of their children, some of the babysitters have quit their jobs and the difficulties of working parents have increased. It is an expected result that such stressful life events cause an increase in the anxiety levels of individuals.

High, moderate, and positive correlation was found between stress, anxiety and burnout levels of healthcare professionals and their quality of life; high, moderate, and positive correlation was found between stress and trait anxiety, burnout; high and negative correlation was found between stress and quality of life. A moderate and positive correlation was found between trait anxiety and burnout, and a high and negative correlation was found between trait anxiety and quality of life. Again, a high and negative correlation was found between burnout and quality of life.

High, moderate, and positive correlation was found between stress and trait anxiety, burnout of healthcare workers, and a high and negative correlation was found between stress and quality of life. A moderate and positive correlation was found between trait anxiety and burnout, and a high and negative correlation was found between trait anxiety and quality of life. Again, a high and negative correlation was found between burnout and quality of life.

In the study conducted with 376 healthcare workers in Italy, one of the countries most affected by the COVID-19 outbreak, the burnout levels of employees were found to be very high (Barello et al., 2020).

In the study conducted with 1,422 healthcare workers during the COVID-19 epidemic in Spain, a total of 56.6% of their employees’ post-traumatic stress disorder symptoms were observed, in 58.6% anxiety disorder, in 46% depressive disorder, and in 41.1% emotional burnout (Luceño-Moreno et al., 2020). These findings support our study.

## Limitations

Furthermore, there is a high proportion of women compared to men. Other studies have shown the same limitation (Gallè et al., 2020; Zhong et al., 2020). In this case, one of the main reasons for this difference is that, in many positions, such as nurses and nursing assistants, the majority of the positions are occupied by women. Another limitation has to do with the cross-sectional design of the study: the pandemic has not yet finished and its influence on mental health cannot be reflected in this research, so it would also be advisable to carry out a longitudinal study that evaluates the evolution over time of the symptoms assessed in this work. On the other hand, there has not been a previous situation in Turkey in which there has been a lockdown, and it is likely that after its ending, the levels of experienced symptomatology will be lower. However, during the SARS crisis, other authors have found that the symptoms of psychological problems after the quarantine period of the disease have lasted up to 3 years later. In the long term, the effects of post-traumatic stress disorder, anxiety and burnout will depend on the possible outbreaks of COVID-19. The measures currently being taken in order to adapt the work place to the new situation (such as providing protective equipment or increasing the number of healthcare professionals) are relevant for mitigating these symptoms. If the appropriate actions to protect health care providers are not taken, they may make medical errors in the future, present higher burnout levels associated with depressive symptoms, anxiety, suicidal ideation, have poorer interpersonal relationships or develop substance abuse. Therefore, a follow-up study along the next few months becomes necessary.

## Conclusion and Suggestions

At the end of the research, it was determined that the stress, anxiety, and burnout of healthcare professionals working during the COVID-19 pandemic had a negative effect on their quality of life.

The healthcare system is among the institutions operating under the most challenging conditions during the period of outbreaks like pandemic which affects the whole world and leads to deaths. During pandemics that affect the society in terms of socioeconomic and mental aspects, the mental health of healthcare teams, who undertake a heavy social and work load, is affected by this situation.

One of the issues that countries should be prepared for in possible future outbreaks such as COVID-19 is taking necessary protective and supportive mental-social measures to protect the mental health of healthcare teams working at the front line as well as their physical health so that they can provide a functional service. In line with this purpose, the following suggestions have been made:

• In order to reduce the mental damage of COVID-19 among healthcare workers, mental health intervention teams can be organized, brochures can be prepared, and a range of mental services including counseling and psychotherapy can be provided to employees and their families.
• Similarly, video interview programs, stress management programs, and group programs aiming at communicating, talking, sharing experiences, and expressing fears and hopes at the end of a working day can be created in order to provide individual mental support for employees in COVID-19 units.
• More healthcare professionals can be recruited in order to reduce the pressure and workload on healthcare professionals.
• One of the most troubling issues during the pandemic was the inconveniences in accessing appropriate protective equipment. Personal protective equipment can be provided for healthcare professionals.
• Working hours can be re-planned by planning the need for rest of the healthcare professionals and creating working and resting environments that will ensure that not only the risk of infection but also other risk factors arising from insomnia and fatigue are taken under control.
• Supportive administrative practices are suggested to be carried out in order to reduce the stress and anxiety levels of healthcare professionals.
• Regardless of whether future outbreaks are the result of natural or man-made events such as bioterrorist threats or not, it is important to recognize gaps in policies and practices and to develop comprehensive strategies and make investments in order to manage and alleviate infectious disease outbreaks.

## Data Availability Statement

The original contributions presented in the study are included in the article/supplementary material, further inquiries can be directed to the corresponding author.

## Author Contributions

All authors listed have made a substantial, direct and intellectual contribution to the work, and approved it for publication.

## Conflict of Interest

The authors declare that the research was conducted in the absence of any commercial or financial relationships that could be construed as a potential conflict of interest.
